# Technology challenges in a higher education institution: Student nurse experience

**DOI:** 10.4102/hsag.v30i0.2749

**Published:** 2025-05-31

**Authors:** Agnes Makhene, Sanele E. Nene, Miyelani Mhlongo

**Affiliations:** 1 Department of Nursing, Faculty of Health Sciences, University of Johannesburg, Johannesburg, South Africa

**Keywords:** challenges, higher education, student nurse, technological resources, experience

## Abstract

**Background:**

Since the coronavirus disease 2019 (COVID-19) pandemic, the world has faced many challenges, including lockdowns and disruptions to various nursing education programmes. Consequently, higher education institutions (HEIs) were compelled to transition to online learning to continue teaching and learning activities. Nursing students had to cope with the abrupt shift from traditional to Internet-based learning platforms. Some student nurses reported significant challenges regarding technological resources, Internet access and loadshedding, a phenomenon specific to South Africa.

**Aim:**

This article aimed to explore and describe technological resource challenges as experienced by student nurses in a HEI in Gauteng.

**Setting:**

The setting was an institution of higher learning offering nursing programmes leading to undergraduate and postgraduate qualifications.

**Methods:**

A qualitative, descriptive, exploratory and contextual design with a phenomenological approach was used. The population consisted of postgraduate students who were exposed to online learning. A purposive sampling method was used. Unstructured, face-to-face and virtual individual phenomenological interviews were used to collect data. Data saturation was reached at the tenth (*N* = 10) participant. Braun & Clarke’s thematic data analysis method was used. Lincoln and Gubas’s strategies and authenticity were used to ensure trustworthiness while observing ethical considerations throughout.

**Results:**

Four themes were identified under the technological resource challenges as experienced by student nurses: loadshedding, connectivity, digital skills and not having a computer.

**Conclusion:**

Online teaching and learning is an essential phenomenon in the 4th Industrial Revolution era; however, students face different challenges that nurse educators need to consider.

**Contribution:**

This study contributes to the information on how the online experience of diverse students who have challenges can be improved.

## Introduction

### Background

The extent of the disparities among nursing students has become apparent in the post-COVID 19 (coronavirus disease 2019) pandemic era. The desire for higher education to switch to online teaching and learning modalities in the latter half of 2020 to 2022 increased the digital divide. In some areas around South Africa where students live and the healthcare facilities where they were employed, it was found that the infrastructure for dependable Internet connectivity was insufficient or unstable. This interferes with web-based resource access, video conferences and online learning. Rony, Parvin and Ferdousi (2024:6) asserted that nurse educators need sufficient training and technical assistance to efficiently employ and incorporate technology into nursing education. Ineffective use of technology and troubleshooting technology problems also result from a lack of training. However, many nursing education institutions around South Africa are aware of the cost associated with developing and maintaining technological solutions. Financial restrictions may prevent certain nursing programmes from purchasing equipment, software and other technological resources needed for efficient online teaching and learning. Research has also shown that these challenges are found globally, especially in rural and underdeveloped areas. Integrating and using technology into the curriculum calls for certain infrastructure, knowledge and resources. Das and Madhusudan (2024:143), in support of this, agree that nursing educators must keep abreast of emerging technologies and the challenges they present to effectively adapt their curricula to incorporate new technological tools and practices.

Attendance of classes from home, using technology in teaching and learning offers flexibility, reduces travel expenses and fosters family time. The researchers’ experience was that the students found the home setting was less suitable for teaching and learning, and that it could be disturbing. The advantages of using technology in the classroom and for theoretical aspects of nursing education are not the only benefits of e-learning (Subedi et al. 2020:73). Furthermore, in their study, Subedi et al. (2020:75) stated that students asserted that technology gives them access to a variety of websites that help them make wise clinical decisions on-the-spot when placed at clinical facilities for work-integrated learning (WIL).

In addition, it has been demonstrated that integrating technology into healthcare environments fosters creative thinking that leads to wise therapeutic decisions. The use of virtual reality (VR), augmented reality (AR), cloud computing and social media through platforms such as Twitter, Dropbox, Google Apps and YouTube has become the norm in modern education and has greatly enhanced the quality of higher nursing education (Mackay Anderson & Harding 2017:3; Márquez-Hernández et al. 2020:5). Such technologies have become an integral aspect of learning these days. While technology-assisted teaching and learning in nursing education offers considerable benefits, there are obstacles that still hinder the advancement of this rapidly developing educational method. According to Maatuk et al. (2022:21–38), a lack of face-to-face engagement, connectivity problems, hardware and software, data security and privacy issues and other difficulties prevent technology from being used effectively in teaching and learning. The absence of in-person interactions with peers and instructors is one of the primary issues. Some students believe that traditional classroom instruction offers more support and involvement than online learning. They believe that the person-to-person interaction with lecturers and peers in the traditional classroom is far more better as it involves human interpersonal relation and interaction as opposed to online interaction (Castro et al. 2022:79; Innab et al. 2022:30–31; Innab & Alqahtani 2023:2554). The preceding citations support the challenges that were experienced by the participants in this study.

### Aim

This article aimed to explore and describe technological resource challenges as experienced by student nurses in a higher education institution (HEI) in Gauteng province, South Africa.

## Research methods and design

A qualitative, exploratory, descriptive and contextual design with a phenomenological approach was used. The study took place at an institution of higher education in a nursing department where several postgraduate and undergraduate nursing programmes are offered.

### Setting

The research took place at an institution of higher learning offering nursing education programmes leading to various undergraduate and postgraduate qualifications. The HEI was chosen as it was also subjected to moving from contact teaching and learning to using technology to give online classes and assessments during the lockdown amid the COVID-19 pandemic era.

### Population and sampling

The population for the study involved postgraduate students who were exposed to online learning at a time when they could not be prepared for this transition. The targeted population for this study was the nursing students who studied through online learning between 2020 and 2021. This grouping of students was seen to be potential participants who will be able to provide the researchers with relevant information. The students were recruited during a Microsoft Teams meeting to which they were invited to inform them about the study and its objectives. A non-probability purposive sampling method was used to select participants who provided rich and in-depth information on their experiences of technological resource challenges (Grove & Gray 2019:107). A virtual meeting with the participants was held to brief the potential participants on the study and related objectives. The participants who were interested on participating signed consent forms that were e-mailed to them. The sample size of 10 participants (*N* = 10) was determined by data saturation. The inclusion criteria included postgraduate students registered in the post-basic BCur (Ed et Admin) programme, exposed to teaching and learning using technology for online teaching and learning and willingness to participate. On the other hand, undergraduate students and those not willing to participate were excluded.

### Data collection

Unstructured, face-to-face and virtual individual phenomenological interviews using Microsoft Teams were used to collect data until saturation was reached. The virtual interviews were conducted for the participants who could not be accessed because of COVID-19 restriction.

Data saturation was reached at the tenth (*N* = 10) participant when no new information came up (Polit & Beck 2020:60). English was used for the interviews as all participants had a fair command of the language as it was used as a medium of instruction in their teaching and learning. The central question that was posed to the participant was: ‘Please share your technological challenges as experienced during the COVID-19 pandemic’. Follow-up questions were asked based on the participants’ responses using communication strategies of paraphrasing, reflection, probing, bracketing and silence. The two researchers, who had no relationship to the participants, also kept field note that enriched the data analysis. The interviews were recorded following the participants’ consent. The audiotapes helped with verbatim transcription of the interviews.

### Data analysis

Thematic data analysis method was used (Braun & Clarke 2006:78). The researchers familiarised themselves with the data by reading and re-reading the transcripts with an aim of generating initial codes. While reading the transcripts, all the interesting information was highlighted as familiarisation with data allowed the richness of the initial findings to emerge. Re-reading the transcripts carefully and coding all the data aimed to find out the patterns and relationships between and across the entire data sets. Data were organised in a meaningful and systematic manner. Data were coded to reduce them into small chunks of meaning. The researchers reviewed, modified and developed preliminary themes that were identified and assessed if they made sense. This phase helped them to figure out the type of themes that might emerge through the data to avoid the influence of the authors’ prior knowledge and experience in the field. The codes were then analysed considering how they could be combined to form an overarching theme. The researchers returned to and re-read all the transcripts before clustering codes according to the themes. Thus, the transcripts were re-read, and different codes were combined into potential themes, collating all the relevant coded data extracts within the identified themes. All the themes (master themes, main themes and sub-themes) were intentionally brought together with the aim of refining those that were initially grouped and presenting them in a more systematic way (LoBiondo-Wood & Haber 2018:576–577).

### Trustworthiness

Lincoln and Guba’s (1985:289–331) and Polit and Beck’s (2018:537) strategies, namely credibility, dependability, transferability, confirmability and authenticity, were used to ensure trustworthiness of the research. To ensure credibility, the researchers used probing and paraphrasing to seek clarity about participants’ responses and member checking to solicit the participants’ perspective regarding the credibility of findings and the interpretations thereof (Creswell & Poth 2018:341). Dependability was established through the description of steps followed in the research to enable other researchers to replicate the study to similar contexts and determine whether the same findings will be found. Rich, detailed and descriptive information about the methods used in this research was provided to ensure transferability. The researchers reduced bias using reflexivity by allowing the participants to fully express themselves without contributing to their statements or responses (Polit & Beck 2020:121). Authenticity was ensured using fair and faithful processes during data collection, analysis and interpretation of the data. To this end, interviews were audio recorded to capture the participants’ thoughts or expressions regarding their experiences (Polit & Beck 2018:537).

Participants signed informed consent. Their confidentiality and privacy were observed throughout the study. The names of the participants were not used during interviews nor mentioned anywhere in the research documents. The information was kept in a password-protected file, and only the researchers had access to the data. The participants were made aware of their right to withdraw their participation at any stage of the research with no consequences for them.

### Ethical considerations

Ethical approval to conduct the study was granted by the University of Johannesburg Faculty of Health Sciences Research Ethics Committee (clearance no.: REC-1590-2022).

## Results

Sociodemographic characteristics of participants who included males and females, whose ages ranged from 32 and years 55 enrolled in the post-basic BCur (Ed et Admin) programme. Participants experienced online learning negatively as evidenced by identified themes (Table 1) that included loadshedding, connectivity, digital skills and not having a computer. These are demonstrated in the quotes as outlined in the following sub-sections.

**TABLE 1:** Themes and sub-themes.

Theme	Sub-themes
1. Loadshedding	1.1 Lack of electricity 1.2 No alternative energy production
2. Connectivity	2.1 Lack of internet connectivity 2.2 Lack of data
3. Digital skills	3.1 Inability to type quickly
4. Not having a computer	4.1 Use of mobile phone

### Loadshedding

Students had a challenge of loadshedding whereby electricity went off while they were in an online class and Internet connectivity was hampered.

In this context, the participants cited the following:

‘I’d wake up at midnight to pray to God to help me with no loadshedding and help me to write what the lecturer will understand even if I didn’t finish. The electricity story was frustrating because we were helpless as you would be kicked out and not be able to log on again.’ (P2, Female, 45 years)

‘My worst experience was being worried about being kicked out by loadshedding and not given another attempt to write the assessment [*Saddened voice*].’ (P9, Male, 46 years)

‘It was frustrating remember also because of lockdown access to the campus was limited and restricted where even if there was loadshedding the university generator will kick in for energy.’ (P7, Female, 50 years)

The students cited issues with the lack of electricity because of loadshedding, a phenomenon experienced by South Africa. This made it impossible for them to attend the online class leading to them losing class time. Sometimes this would happen during an assessment, which caused a lot of frustration for the students as they were not sure if they will be afforded a second opportunity to write the assessment. Some students did not have an alternative source of energy that could be used during electricity outages because of loadshedding.

### Connectivity

The students encountered challenges with Internet connection. Inability to connect to the Internet posed a challenge in connecting to online classes and writing assessment online.

One student said:

‘[*M*]y biggest challenge was the internet. Some of us don’t Wi-Fi connection at home unlike when we are on campus we use the university Wi-Fi and don’t have to pay for it. This online thing was frustrating. I was not even sure I will pass the course.’ (P3, Female 50 years)

Another said:

‘Online learning was not cost effective for me because I had to buy data for connecting to the internet, which became expensive for me. Remember some of us don’t have money [*Brief silence*].’ (P1, Female 39 years)

Students were challenged as far as connectivity is concerned as most did not have Wi-Fi connection or the Internet. It was clear that online learning also posed a challenge financially as student had to buy data to be able to connect to the Internet or go to an Internet café where they had to pay to use their equipment and services to connect to the online class.

### Digital skills

The lack of computer skills on the part of the student led to challenges with logging on and typing. Participant cited that it was difficult to follow conversation, engage during lessons and write assessments because of their lack of technological skills.

The participants in this context stated:

‘Initially, I was apprehensive about online learning since it was new to me, and I was not very proficient with computers, especially compared to younger generations. This made me feel scared and uncertain about whether I could cope with the demands of online learning.’ [*Brief silence*] … I started having anxiety especially when I must write assessments, and once I do that, I’d start excessively explaining, and I will lose the context.’ (P7, Female, 50 years)

‘… My biggest challenge was being unable to type quickly and submit assignments online. Remember some of us were never exposed to technology and its use before at school even at home. Our parents did not afford buying even computer at home for use by everyone.’ (P6, Female, 54 years)

Other participants recommended the following to address the lack of digital skills of students:

‘It would be beneficial for students to receive computer training before. This is because some tutors post class links while others do not. Many students struggle to locate the links online, even though they are expected to do so as tertiary students.’ (P5, Female, 38 years)

‘So I think the lecturers must keep on consulting with the students because I feel like the lecturers just give you the work, and they don’t come back to ask if you are ok.’ (P4, Male, 45 years)

Most of the students in the BCur (Ed et Admin) programme were matured students coming from previously disadvantaged background with no digital skills. Many of them struggled to type quickly especially during assessments where they were timed, creating frustration and discouragement.

### Not having a computer

Students also had a challenge of not owning a computer, which created an obstacle when they are expected to attend class, write assignments and submit assignments digitally.

One participant expressed the following:

‘[*F*]or me the biggest challenge was not having a computer and having to use my mobile phone to do everything, even writing assessments. This was very frustrating as I was typing slow and often not finish assessments. I experience a lot of anxiety and stress when we had to write assessments and even failed a few of them [*sounding very sad*].’ (P5, Female, 38 years)

A participant reported that:

‘[*J*]a it was challenging. I do not own a computer because I cannot afford one. Imagine writing an assessment on the phone and typing with one finger, [*pause*] it took long and I would lose my work in between. It was just sad and made me feel so inadequate and lecturers were not sympathetic [*sounding very sad*].’ (P8, Female, 55 years)

Another participant stated that:

‘Attending class while at home was very challenging for me, although it was possible to do work at one’s pace and time, it wasn’t the best option for me as I did not have a computer and had to use my mobile phone.’ (P10, Female 40 years)

A different participant indicated that:

‘[*O*]nline learning forced me to be on my own, and not having a computer added to my frustration, as I had to use my phone or go to the internet cafe and that really affected my performance.’ (P1, Male, 32 years)

It was clear that during the switch from contact to online classes, students faced several difficulties. Some students did not even own a computer and had to use their mobile phones to log onto class, which posed a challenge especially when writing assessments and keeping to time allocated for the assessment and saving or submitting work.

## Discussion

The research question of this study exposed the challenges that the participants faced with online teaching and learning during the COVID-19 era as evidenced by the above-stated citations. Loadshedding mainly led to other factors that posed challenges for students. In addition to creating social and economic difficulties, loadshedding also interfered with students’ educational opportunities and presented several other difficulties that raised the stress levels for students. Not only is the issue interfering with the teaching and learning process, but it also hinders with the operation of online courses and the availability of educational resources (Hlatshwayo 2022). Loadshedding also raises serious health issues for students that involve agitation, boredom and tension, which can hamper their academic performance and squander study time (Mthanti 2023:165). Maharaj (2023:64), on the other hand, proposes that there are online teaching strategies that can establish a conducive and encouraging environment for student inquiry, thereby increasing their understanding of the subject matter and stimulating critical and enthusiastic thinking as well as reflection on students’ learning.

According to Khoza (2024:2148), to ensure that crucial tasks such as teaching and learning are not impacted by loadshedding, educational institutions and other organisations need to use renewable energy to enable connections if there is loadshedding. Internet connectivity also impacted the connections between lecturers and students, which hampered the quality teaching and learning (Rahman 2021:82; Turnbull, Chugh & Luck 2021:6410; Wopula, Nene & Nkosi 2022:6). Singh et al. (2021:6) argued that challenges encountered when utilising technology for teaching and learning include network-related problems such as audio virtual disparities, session interruptions because of unexpected network logging out and continuous buffering. According to Sabeghi et al. (2021:e114569), one of the most frequent complaints raised by students enrolled in online courses is technological difficulties. They contend that many students might lack the technical know-how required for online learning activities, and that simply being able to use and navigate social media with other online platforms does not imply that a person is proficient with educational technology.

Compared to in-person classes, students who learn online feel not only less connected to their teachers and peers but also more frustrated and anxious. It was found that there is value in social interaction and communication channels in online learning; however, it was pointed out that the absence of social experiences has detrimental effects on students’ educational experiences and that they could worsen if these channels are not appropriately put in place in online learning environments (Luan et al. 2020:9; Procentese et al. 2020:10; Suliman et al. 2021:4). Other issues include inadequate connectivity and a lack of technological literacy (Nene & Hewitt 2024:2; Suliman et al. 2021:3; Uprichard 2020:275).

However, Coman et al. (2020:22) attested that most students favour in-person instruction, as well as blended and hybrid learning models that incorporate peer and teacher interaction in lecture halls. Moreover, students’ dissatisfaction and likelihood of dropping out are increased by the unfavourable reactions by the marginalised university authorities and critics, which demotivates them.

The widening inequality gap in South Africa is mirrored in the country’s digital divide. According to Rotas and Cahapay (2020:150), many students lack the technological resources they need for online learning, and loadshedding and weak or absent Internet connectivity further exacerbate the problem. As a result, students from poor socioeconomic and rural backgrounds lack a supportive learning environment (Maatuk et al. 2022:38). The feasibility of using high technology is questioned if students are going to be disadvantaged (Gloria & Uttal 2020:145). As Internet service is often interrupted during a power outage, this means students will lose both electricity and Internet connection at the same time. This affects the teaching and learning network. Similarly, this challenge also concerns lecturers who need electricity and the Internet to organise online learning. The digital divide and the lack of current technology are experienced more by students from lower-income families who have limited or no access to online learning (Hasan & Bao 2020:6).

Disparities in socioeconomic conditions are exacerbated by the lack of resources, including access to educational technology, the Internet and infrastructure to implement online learning among students from poor backgrounds (Czerniewicz 2020:n.p.). Principles such as social justice require that nurse educators individually assess and support students at risk of disadvantage because of technological disparities. Access to technology and digital literacy will greatly influence the ability of nursing graduates to use technology to meet health needs of future populations. According to Siemens (2005:n.p.), the theory of connectivism promotes group collaboration and dialogue, considering different perspectives and points of view in decision making, problem solving and information understanding during online teaching and learning. This can be difficult for students who face technology challenges. A key aspect of this theory is that technology should be at the forefront of teaching and learning in the digital age. Thus, the digital age challenges require both the teachers and students to rethink the role of technology in the development of knowledge. This theory suggests that the new technology such as social media, online networks, blogs or databases can support new-age learning (Barrot, Llenares & Del Rasario 2021:7328).

In addition, having the technological know-how to operate with a variety of digital platforms, applications and spaces is crucial for offering effective online education. Therefore, students must be taught how to use various online learning platforms and learning management systems (Moyo et al. 2022:35). However, because of the high cost of the Internet, equipment and poor network quality in certain areas in the country, students feel mentally worthless and this accelerates the process of leaving their studies before they finish (Saha, Dutta & Sifat 2021:174). Rahman (2021:72, 88–89) added that long interactive online classes present more challenges than just device compatibility. It is important for students to have access to electricity and a substantial amount of mobile data, in addition to device compatibility. Similar to other academic establishments, nursing education institutions ought to address the growing digital gap that contributes to learning disparities and social injustices (Moyo et al. 2022:30). Huda (2023:88) argues that educational institutions ought to establish a second opportunity on campus in case certain obstacles are not immediately resolved.

The rapid change by universities to online teaching and learning disadvantaged many students because of the lack of resources. To this day, a permanent change to online learning is still out of reach for institutions of higher learning, especially in the South African context, where historically underprivileged universities continue to face glaring differences in infrastructure and Internet connectivity. The COVID-19 pandemic exposed and highlighted these divisions, which have presented a serious obstacle to the goal of achieving inclusive and equitable higher education since 1994 (Bhaumik & Priyadarshini 2020:246–248).

### Implications

Nursing education faces several technological challenges that can impact the delivery of training and education to aspiring nurses. One of the key challenges is access to technology. Not all nursing students may have equal access to technology, such as computers, high-speed internet or mobile devices. This digital divide can hinder their ability to participate fully in online learning activities or access educational resources. A lack of face-to-face engagement, connectivity problems, problems with hardware and software, data security and privacy issues and other difficulties prevent technology from being used effectively in teaching and learning. Nursing education involves handling sensitive patient information and complying with privacy regulations. Ensuring the security of digital platforms and protecting educational data from breaches or unauthorised access is also crucial but can be challenging. Different nursing programmes or institutions may use various technology platforms and systems, leading to inconsistencies in the learning experience. Standardising technology across educational institutions can help create uniformity and streamline the learning process. Educational institutions and the government need to be reoriented towards a developmental agenda that requires a significant restructuring of funding models to bridge the digital divide in our educational institutions to afford all students equitable quality education where all have equal opportunities and resources. Further research should be done on how the digital divide gap can be bridged and how students can be equipped to empower them with digital skills. Furthermore, a transformation agenda that prioritise research that addresses the needs of marginalised communities should be emphasised, especially students.

### Limitations

The researchers could not reach a wider group of students who have been exposed to online learning in different programmes.

## Conclusion

Nursing educations institutions can enhance a positive online learning experience for all students, irrespective of their historical background, economic status and Internet connectivity by using recyclable energy, providing basic computer training and making computer laboratories and data accessible to all students.
